# Evaluation of class participation in non-face-to-face CPR training for medical students

**DOI:** 10.1371/journal.pone.0278273

**Published:** 2022-12-01

**Authors:** Young Shin Cho, Hye Ji Park, Daun Choi, Hang A. Park, Sola Kim, Ju Ok Park, Soon-Joo Wang, Choung Ah Lee

**Affiliations:** 1 Department of Emergency Medicine, Soonchunhyang University Seoul Hospital, Yongsan-gu, Seoul, Republic of Korea; 2 Department of Emergency Medicine, Dongtan Sacred Heart Hospital, Hallym University, Hwaseong-si, Gyeonggi-do, Republic of Korea; 3 Hallym Dongtan Simulation Center, Hwaseong-si, Gyeonggi-do, Republic of Korea; Fondazione IRCCS Policlinico San Matteo, ITALY

## Abstract

**Background:**

Cardiopulmonary resuscitation (CPR) education requires that learners practice key skills to promote mastery. Our aim in this study was to evaluate differences in post-education performance and class participation during CPR training between face-to- face (FF) and non-face-to-face (NFF) learning formats.

**Methods:**

This was a randomized controlled study of third-year medical students from two university hospital, allocated to either the FF or NFF format for CPR education. The learning scenario addressed single-person CPR, consisting of chest compression only, and excluded breathing. The Kahoot! application was used for NFF. Between-group comparisons for class participation and CPR skills were based on video recordings.

**Results:**

Seventy students participated in our study, with 35 randomly allocated to the FF and NFF groups. There were no between-group differences in terms of age, sex, previous basic life support training, and willingness and confidence in performing CPR. Compared to the FF group, the NFF group demonstrated significant differences during CPR, including fewer calling for assistance and using of defibrillator (p = 0.006), as well as fewer checking for breathing (p = 0.007), and fewer counting during chest compression (p = 0.006). Additionally, < 30% of learners in the NFF group completed rhythm analysis after the last defibrillator shock delivery and resumed immediate chest compression (p < 0.001). All students in both groups passed the post-training assessment.

**Conclusion:**

Class participation in NFF learning was lower than that in FF learning. Although the post-education evaluation in the NFF group was not inferior, efforts on promoting active participation in NFF learning are required.

## Introduction

Since the onset of the coronavirus disease 2019 (COVID-19) pandemic, major changes in educational methods have been required [1] to shift from face-to-face (FF) training to a non-face-to-face (NFF) format [2]. Physicians with academic responsibilities have used various NFF education formats to reduce academic burden and improve students’ knowledge and skills [3]. However, multiple factors are barriers to distance learning, including best methods to facilitate general learning and learning in practice, absence of a systematic approach for NFF and the integration of e-learning into curricula, poor student motivation and expectation, and lack of information on efficient use of available technologies and communication skills [4].

Cardiopulmonary resuscitation (CPR) training is a compulsory curriculum for medical students [5]. The application of NFF methods for CPR education is a specific issue of interest in medical education as CPR instruction allows learners to practice key skills until they attain mastery [6]. CPR education requires that the availability of resources, such as equipment for practice, and educational content and training method be considered as an integral whole [7]. With the need for a shift to NFF formats of CPR education, different educational methods have been studied, including the use of video-based and virtual reality training, as well as e-learning modules [8]. Previous studies have shown that NFF education may not be inferior to FF [9, 10] but may be insufficient for skill mastery [11]. For effective NFF learning, the self-learning ability of students should be evaluated and improved [12]. Active class participation is one of the factors that has been used to evaluate the effectiveness of self-directed learning, considering its importance for successful learning [13]. Our aim in this study was to evaluate class participation during CPR training between FF and NFF formats.

## Materials and methods

### Study design and participants

This was a randomized case-control study of an educational intervention for CPR, performed between June 2020 and September 2021. The study sample included 70 third-year medical students recruited from two universities in South Korea. All participants were informed that video recording would be used to monitor class participation and CPR skill performance.

### Sample size and randomization

Sample size calculation was performed prior to the trial, based on the data for distance training presented by Han et al. [14]. Twenty-six participants in each group were needed at a level of significance at 5% (alpha = 0.05) and a power of 80% (beta = 0.2). Based on a presumed exclusion rate of 25%, 70 learners were included, 35 randomly allocated each to the FF and NFF learning groups. Allocation was performed using block randomization, with a 1:1 allocation ratio.

### Training course

Educational content and methods for CPR for both groups were based on the recommendations of the Korean Association of Cardiopulmonary Resuscitation (KACPR). The KACPR guidelines underline the necessity for hands-on practice, recommending the use of feedback equipment and practice while watching (PWW) to guide skill mastery. Adhering to the guidelines to lower risk of COVID-19 spread, a single-person rescue CPR scenario was used with chest compression only while excluding breathing. Accordingly, non-technical skills, such a teamwork, communication, and debriefing between learners, were not included. The same educational content was used for NFF learning; instructions for the use of the mannequin for CPR, with feedback provided during chest compression practice were added.

The course process for basic life support (BLS) training for both groups is shown in Table 1. For the FF format, training was performed under instructor supervision, with five learners per instructor. Theory and skill practice were conducted using video clips. For the NFF format, the interactive component was provided using the Kahoot! application [15]. Theory lecture and videos for PWW were used to explain and demonstrate each step of the CPR process. The NFF format was structured to allow learners to progress at their own rate. On acquisition of knowledge for each step, they had the opportunity to complete practice with the mannequin. To minimize contact between learners, one mannequin per person was used.

**Table 1:** Course process of the face-to-face and non-face-to-face formats for basic life support training.

	Face-to-face learning	Non-face-to-face learning
Training content	Assessment and Performance (10 min) • Check patient responsiveness and activate the emergency response system. Instructional mode—theory lecture and PWW • Check patient’s breathing and pulse (PWW)	
	Chest compression instructional mode—theory lecture (5 min)	
		Instruction on how to use the CPR mannequin and feedback devices for practice (5 min)
	Chest compression practice mode (5 min) • 7 cycles of 30 compressions, total of 2 min (PWW)	
	Use of automated external defibrillator. Instructional mode—theory lecture and PWW (10 min)	
	Scenario based integrated practice (10 min)	
Learning resources	Instruction 1:5 instructor to learner ratio	Isolated training room for practice; self-learning on tablet (e-learning)
	Lecture and practice using video clips	Learning platform: Kahoot!
	1 mannequin per learner	1 mannequin per learner
Total training hours	40 min	45 min
Evaluation	• Class participation by checklist through video recording • On-site evaluation for course pass	

PWW, practice while watching

### Measurement

Pre-education questionnaires were distributed to collect the following self-reported information: age, sex, previous CPR training, and willingness and self-efficacy in CPR on witnessing cardiac arrest. Willingness and confidence were evaluated on a 5-point Likert scale by modifying the questionnaire used in the study of Ro et al. [16].

**Fig 1:**
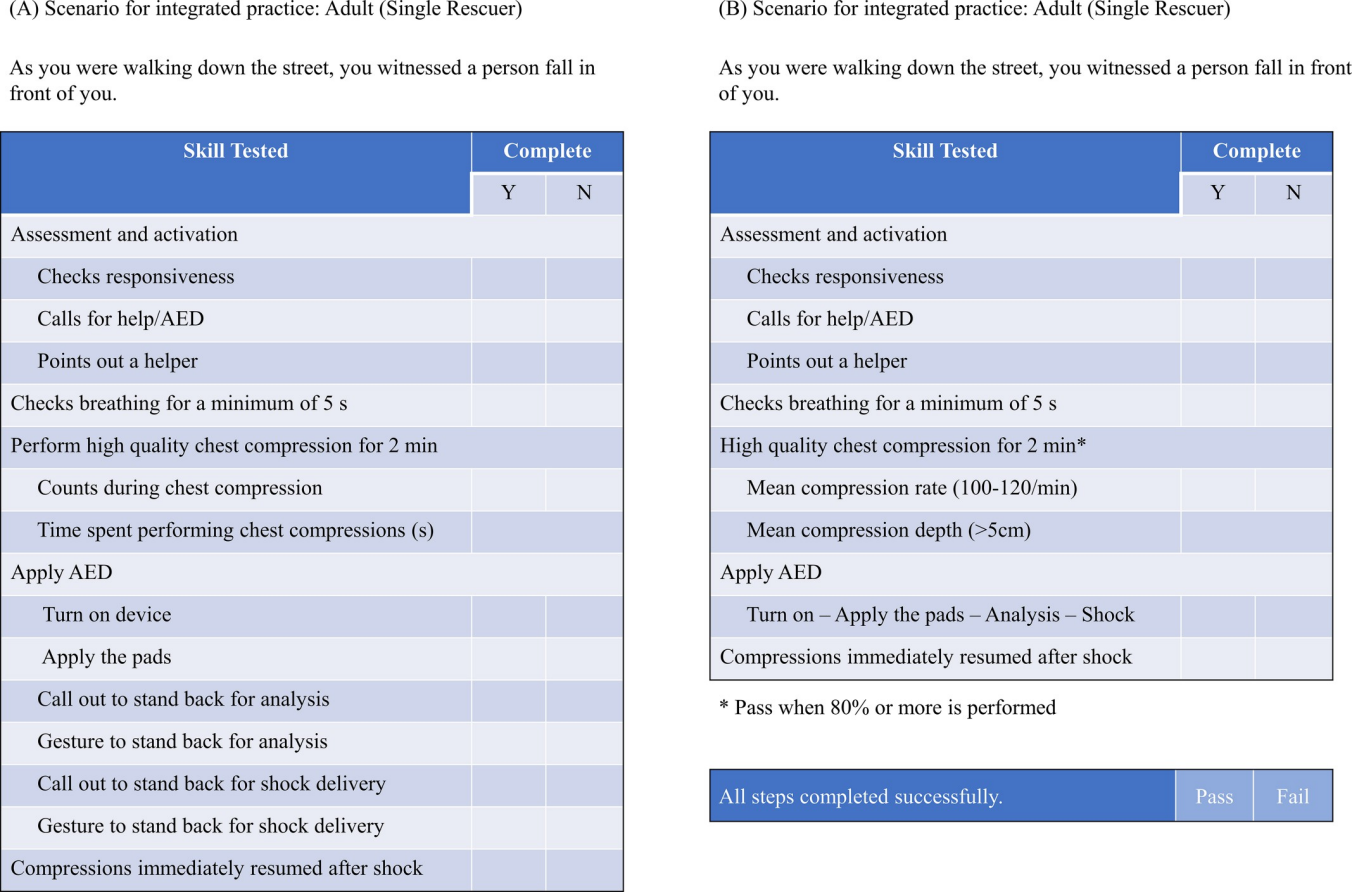
Scenario and checklist of integrated practice for an adult case with a single rescuer. (A) and (B).

The primary outcome was class participation. Class participation was evaluated based on video recording of individuals during FF and NFF sessions. We checked whether the learners actually practiced by following the video for each BLS step. In the chest compression step, the fraction of the time actually utilized for chest compression during the entire video playback time was expressed as percent of the total required time of 120 seconds (Fig 1A).

As a secondary outcome, performance after the education was evaluated according to the checklist (Fig 1B). Whether each step was performed was assessed, and in the chest compression step lasting 2 minutes, it was evaluated whether accurate compression rate (100-120/min) and compression depth (5–6 cm) were adhered to more than 80%. If all steps were appropriate, participants were adjudged to have passed the course.

### Data analysis

Between-group differences were evaluated using a chi-squared test for categorical variables and a Mann-Whitney U test for continuous variables with a non-parametric distribution. Logistic regression and linear regression analysis were performed to evaluate the association between learning methods (FF and NFF) with regard to class participation. As effect size index, Cohen’s d was used to indicate the standardized difference for continuous variables and Cohen’s ω was used to compare the distribution of a categorical variable [17]. We considered the effect size as small when Cohen’s d was less than 0.2, medium when it was approximately 0.5, and large when it was greater than 0.8. As for Cohen’s ω, the effect size was considered as small when its value was smaller than 0.1, medium when it was around 0.3, and large when it was greater than 0.5. All statistical analyses were performed using SPSS (version 25.0; IBM Corp., Armonk, NY, USA).

### Ethics

This study was approved by the institutional review board of Hallym University (HDT 2020-06-023) and all participants provided written informed consent.

## Results

**Fig 2:**
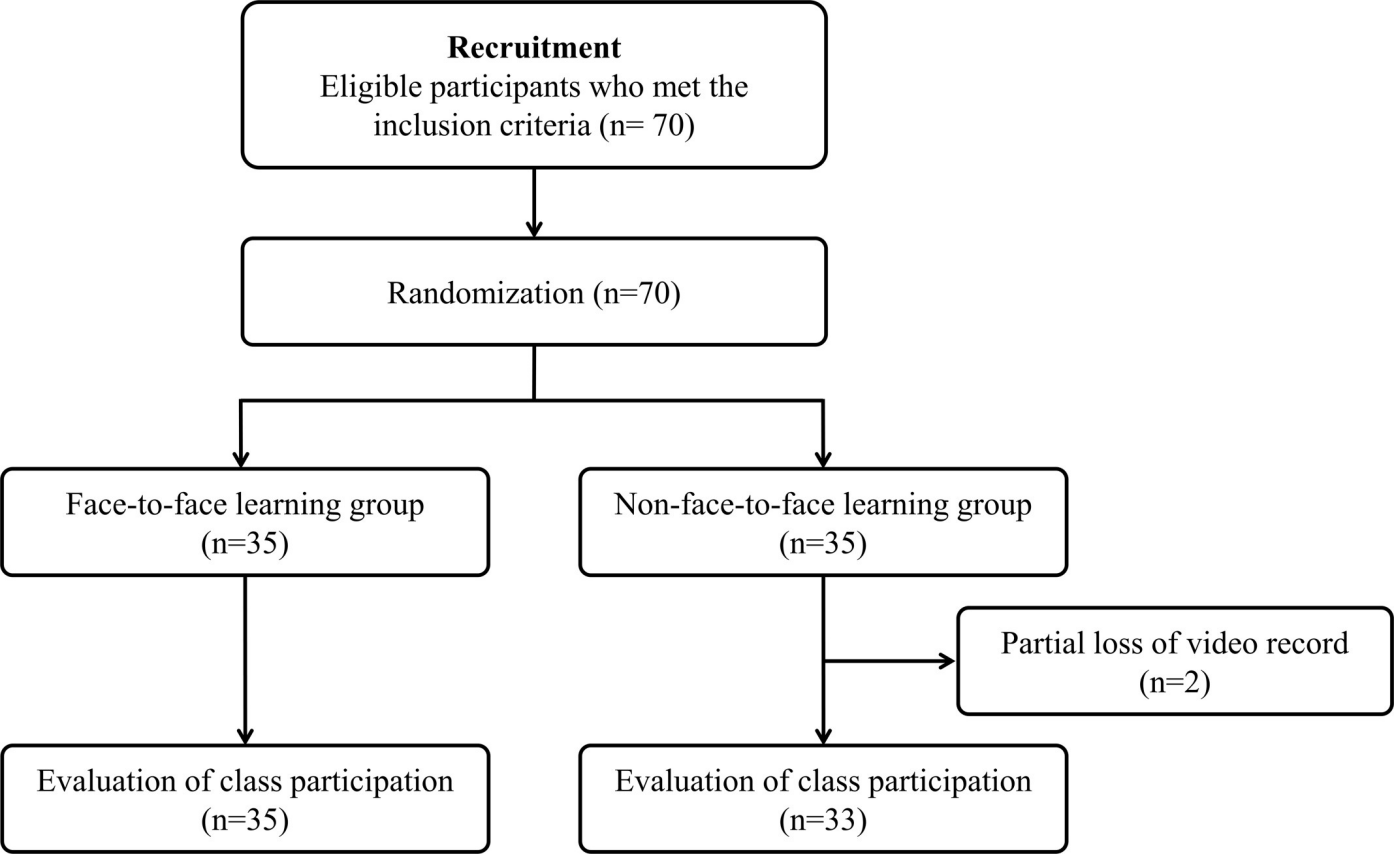
Flowchart for the allocation of participants to the FF and NFF learning groups and video data available for evaluation of class participation and post-training skills. FF, face-to-face; NFF, non-face-to-face.

Of the 70 learners included, the video for two participants in the NFF group was not recorded due to insufficient storage memory; therefore, videos from 33 cases were used in the analysis (Fig 2).

There were no between-group differences in the distribution of learners’ age, sex, and previous BLS training. The willingness to start CPR on encountering a collapsed individual was high before training in both groups, with a willingness score of 4 or 5/5 in ≥ 90% of learners. Regarding confidence in CPR skills (strongly agree and agree), 74.2% and 80.9% in the FF and NFF groups, respectively, reported that they were confident (Table 2).

**Table 2:** Basic characteristics of participants in the face-to-face and non-face-to-face learning groups.

	Total (n = 68)	Face-to-face learning (n = 35)	Non-face-to-face learning (n = 33)	p-value
Age, years	24 (23, 25)	23 (23, 25)	24 (23.5, 26)	0.07
Sex, n (%)				0.42
Male	42 (61.8)	20 (57.1)	22 (66.7)	
Female	26 (38.2)	15 (42.9)	11 (33.3)	
Two or more previous BLS trainings	29 (42.6)	16 (45.7)	13 (39.4)	0.59
CPR willingness				0.29
5 (Strongly agree)	37 (54.4)	16 (45.7)	21 (63.6)	
4 (Agree)	25 (36.8)	16 (45.7)	9 (27.3)	
3 (Neither agree nor disagree)	5 (7.4)	3 (8.6)	2 (6.1)	
2 (Disagree)	0	0	0	
1 (Strongly disagree)	1 (1.5)	0	1 (3.0)	
Performance confidence				0.07
5 (Strongly agree)	34 (50)	13 (37.1)	21 (63.6)	
4 (Agree)	22 (32.4)	13 (37.1)	9 (27.3)	
3 (Neither agree nor disagree)	8 (11.8)	6 (17.1)	2 (6.1)	
2 (Disagree)	3 (4.4)	3 (8.6)	0	
1 (Strongly disagree)	1 (1.5)	0	1 (3.0)	

Data are shown as the median (interquartile range) or count (%). BLS, basic life support; CPR, cardiopulmonary resuscitation

Learners’ participation at each step is reported for both groups in Table 3. Class participation was high on all items for the FF group. By comparison, class participation was significantly lower for the NFF group, including less learners calling for help (p = 0.006) and using AED, checking for breathing (p = 0.007), and counting aloud during chest compression (p = 0.006). Moreover, while all participants in the FF group performed chest compressions for the full required 2 min, only 88% of participants in the NFF performed compressions for the required 2 min (p < 0.001). Furthermore, in the NFF group, less than 30% of learners completed rhythm analysis after the last AED shock delivery and resumed immediate chest compression. Large effect size was observed in all steps except in the ‘turn on device’ and ‘apply the pads’ steps, both of which had a small effect size [17].

**Table 3:** Comparison of class participation between the face-to-face and non-face-to-face learning groups.

	Face-to-face learning (n = 35)	Non-face-to-face learning (n = 33)	p-value	Effect size index
Checks responsiveness	35 (100)	31 (93.9)	0.447	0.5
Calls for help/AED	35 (100)	25 (75.8)	0.006	2.0
Point out a helper	32 (91.4)	24 (72.7)	0.089	0.7
Check breathing for a minimum of 5 seconds	35 (100)	22 (66.8)	0.007	2.8
Counts during chest compression	29 (82.9)	16 (48.5)	0.006	0.9
Fraction of chest compressions (%)*	100 (100–100)	88 (85–92)	< 0.001	1.01
AED				
Turn on device	35 (100)	33 (100)	1.000	0.0
Apply the pads	35 (100)	33 (100)	1.000	0.0
Call out to stand back for analysis	28 (80)	8 (24.2)	< 0.001	1.4
Gesture to stand back for analysis	29 (82.9)	9 (27.3)	< 0.001	1.5
Call out to stand back for shock delivery	28 (80)	7 (21.1)	< 0.001	1.5
Gesture to stand back for shock delivery	30 (85.7)	7 (21.2)	< 0.001	1.8
Compressions immediately resumed after shock	34 (97.1)	8 (24.2)	< 0.001	4.3

* Fraction of chest compressions over the required 2 min duration. Data are shown as the median (interquartile range) or count (%); AED, automated external defibrillator

**Fig 3:**
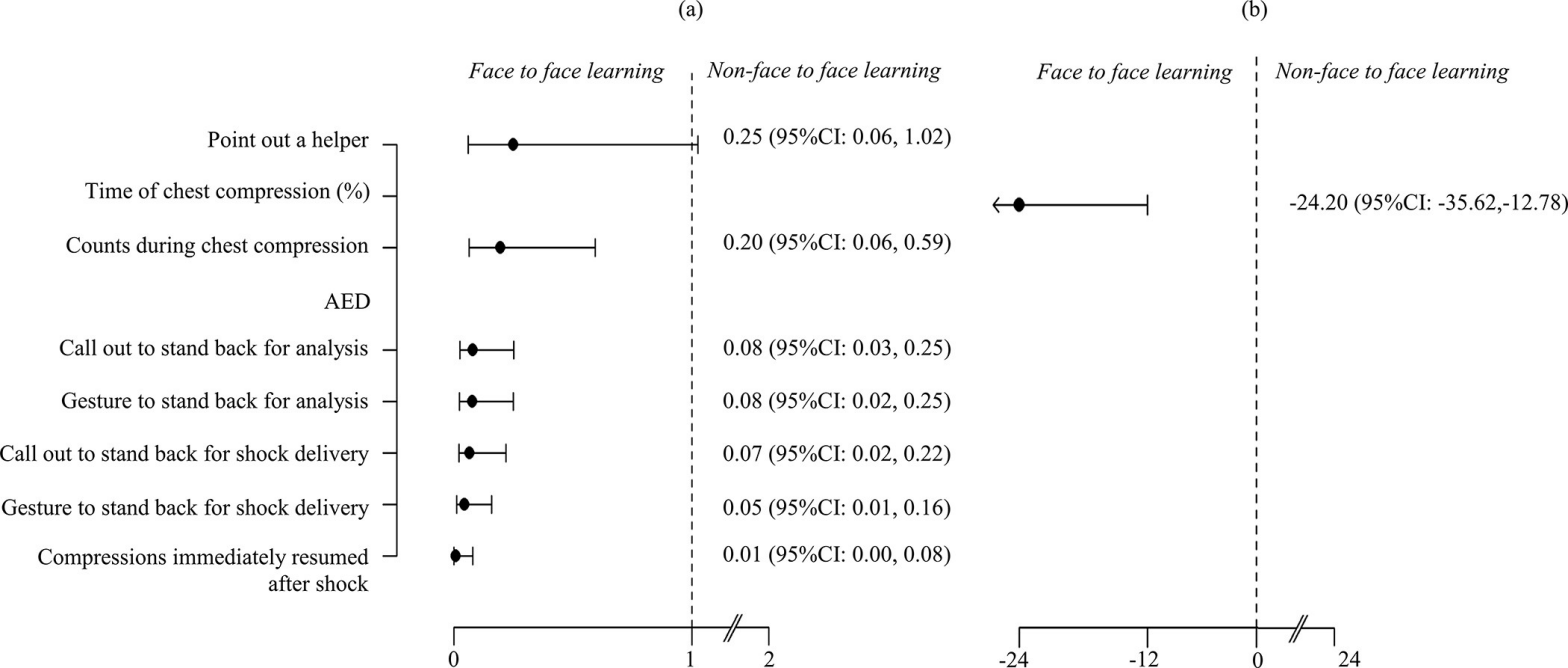
The odds ratio (a) and ß value (b) for learning outcome for the NFF compared with the FF group at each step of the training scheme. CI, confidence interval; AED, automated external defibrillator; FF, face-to-face; NFF, non-face-to-face.

The odds ratios (OR) of learning for each step of the training scheme for the NFF group compared with the FF group are reported in Fig 3. Performance for the following outcomes favored the FF group: checks breathing; counts aloud during chest compression; asks for help and uses AED; and performs chest compressions immediately after external shocking. Moreover, when the fraction of the 2 min during which chest compression was performed was analyzed by linear regression, learning favored the FF over the NFF group: *R*^*2*^, 21.3%, ß, -24.2 (95% confidence interval, -35.6 to -12.8). All learners passed the post-training assessment.

## Discussion

This study focused on class participation between FF and NFF approach to CPR education. Our findings indicate that the participation rate of learners was lower for the NFF than FF approach and that performance outcomes favored the FF over the NFF approach, except for the criteria of responsiveness check, pointing out a helper, and preparing the defibrillator for use. Our findings are important considering the rapid transition to entirely online teaching for medical students due to the COVID-19 pandemic.

Stable accessibility to devices—such as a computer or mobile phone—online learning platforms, and newly developed content have been indicated to yield better learning effects in an NFF than FF environment [18]. NFF learning can provide several benefits to learners, including convenience, time saving, flexibility of scheduling, and improving group interaction through chat function [2, 19]. Moreover, asynchronous NFF learning, as a mode of self-learning, can provide an easier and more effective access to a wider variety and greater quantity of information, as well as providing a personalized approach to learning, with learners having greater control over the educational content, learning sequence, and time spent [2].

Our findings that NFF is not inferior to FF education for CPR knowledge and skills is consistent with those of previous studies [9, 10, 20, 21]. The successful outcome on post-training assessment for both groups might reflect the fact that all students in the study sample had at least one BLS training experience, as they were third-year medical students. Motivation for learning and performance may also have been high considering the volunteer nature of participation. Motivation is an important factor for successful learning [22]. Of note, however, was the significantly lower volume of class participation for the NFF than FF group. In particular, analysis of the cardiac rhythm after the last AED application and immediate resumption of chest compression was performed by < 30% of learners in the NFF group. The difference in class participation between the two groups showed a large effect size in most steps which indicates the practical significance [17].

The lower than expected participation rate for the NFF than FF group may be explained, at least in part, by the following three reasons. The first regards the low interaction within the NFF approach. Croxton found that purposefully designed and engaging interaction tasks played a significant role in learner persistence in online courses [23], where interaction is not only between learners and teachers but also between learners and learners and content [24]. As BLS training is focused on practice, real-time instructor feedback will tend to influence participation in the training, as will observation of peers during practice sessions. Practice on teamwork activities may also be important to promote mastery on specific CPR components, including calling others for help and instruct others to stand back during AED used. Second, lack of monitoring may be a reason for poor participation in the NFF group. Accurate monitoring of learning activities is a key element of self-regulated learning, leading to higher learning achievement [25]. Therefore, examination and evaluation of each learner’s participation in the content and their progress may be important for instructors using the NFF format of teaching. In our study, learners were notified in advance that their participation in education would be evaluated, but no immediate feedback was provided in case of insufficient participation in education. Participation may be further improved by including feedback on learning attitudes, such as providing simple performance feedback [26]. The third factor to consider may be a decrease in participant attentiveness over time, resulting in insufficient learning immersion. From the step after the AED analysis, a significant decline in participation using the PWW method was observed. This is consistent with a previous report of a gradual decrease in participation over time in an e-learning environment [27].

The limitations of our study need to be acknowledged. First is the response bias due to the small sample size, although the number of participants was adequate for sufficient power of analyses. Second, recorded video data were used for analysis. As real-time evaluation of participation was not included, it was not possible to confirm other variables which might have influenced participation in the NFF group, including internet connection failures and device operation errors. Lastly, personal factors which can influence participation in NFF education in particular were not considered, which include problem-solving ability, self-efficacy, and attitude and interest in learning [28]. We did make the assumption that learning attributes were comparable between the two groups.

## Conclusion

Class participation in NFF learning was lower than that in FF learning. Although the post-education evaluation in the NFF group was not inferior, efforts on promoting active participation in NFF learning is required.

## Supporting information

S1 Data (XLSX)
