# Correction: Evaluation of class participation in non-face-to-face CPR training for medical students

**DOI:** 10.1371/journal.pone.0293159

**Published:** 2023-10-16

**Authors:** Young Shin Cho, Hye Ji Park, Daun Choi, Hang A. Park, Sola Kim, Ju Ok Park, Soon-Joo Wang, Choung Ah Lee

There are errors in the Funding section. The correct Funding statement is: This research was supported by the Korean Association of Cardiopulmonary Resuscitation (no. 2021–001) and the Soonchunhyang University Research Fund. The funders had no role in study design, data collection and analysis, decision to publish, or preparation of the manuscript. There was no additional external funding received for this study.
